# Supplementary material for: Digital health interventions for improving access to primary care in India: A scoping review
Source: PLOS Glob Public Health. 2024 May 14;4(5):e0002645. doi: 10.1371/journal.pgph.0002645 (PMC11093327; doi:10.1371/journal.pgph.0002645)
Supplement: S1 Text — (DOCX) [file pgph.0002645.s002.docx]

**Search Strategy:**

Database: Journals@Ovid Full Text <April 30, 2023>, LSHTM Journals@Ovid, Embase Classic+Embase <1947 to 2023 April 30>, Global Health <1910 to 2023 Week 17>, Ovid MEDLINE(R) and Epub Ahead of Print, In-Process, In-Data-Review & Other Non-Indexed Citations and Daily <1946 to April 30, 2023>

Search Strategy:

--------------------------------------------------------------------------------

1 Access*.mp. [mp=ti, ab, tx, ct, sh, hw, tn, ot, dm, mf, dv, kf, fx, dq, bt, id, cc, nm, ox, px, rx, an, ui, sy] (3407375)

2 Accessibility.mp. [mp=ti, ab, tx, ct, sh, hw, tn, ot, dm, mf, dv, kf, fx, dq, bt, id, cc, nm, ox, px, rx, an, ui, sy] (316411)

3 inaccessibility*.mp. [mp=ti, ab, tx, ct, sh, hw, tn, ot, dm, mf, dv, kf, fx, dq, bt, id, cc, nm, ox, px, rx, an, ui, sy] (13670)

4 1 and 2 and 3 (2201)

5 health care.mp. [mp=ti, ab, tx, ct, sh, hw, tn, ot, dm, mf, dv, kf, fx, dq, bt, id, cc, nm, ox, px, rx, an, ui, sy] (3606889)

6 primary health care*.mp. [mp=ti, ab, tx, ct, sh, hw, tn, ot, dm, mf, dv, kf, fx, dq, bt, id, cc, nm, ox, px, rx, an, ui, sy] (245855)

7 health service*.mp. [mp=ti, ab, tx, ct, sh, hw, tn, ot, dm, mf, dv, kf, fx, dq, bt, id, cc, nm, ox, px, rx, an, ui, sy] (1774805)

8 5 and 6 and 7 (82353)

9 Assistive technology*.mp. [mp=ti, ab, tx, ct, sh, hw, tn, ot, dm, mf, dv, kf, fx, dq, bt, id, cc, nm, ox, px, rx, an, ui, sy] (10574)

10 Technology*.mp. [mp=ti, ab, tx, ct, sh, hw, tn, ot, dm, mf, dv, kf, fx, dq, bt, id, cc, nm, ox, px, rx, an, ui, sy] (2062792)

11 Decision support*.mp. [mp=ti, ab, tx, ct, sh, hw, tn, ot, dm, mf, dv, kf, fx, dq, bt, id, cc, nm, ox, px, rx, an, ui, sy] (113898)

12 mHealth*.mp. [mp=ti, ab, tx, ct, sh, hw, tn, ot, dm, mf, dv, kf, fx, dq, bt, id, cc, nm, ox, px, rx, an, ui, sy] (17006)

13 assistive devices*.mp. [mp=ti, ab, tx, ct, sh, hw, tn, ot, dm, mf, dv, kf, fx, dq, bt, id, cc, nm, ox, px, rx, an, ui, sy] (13491)

14 health care support*.mp. [mp=ti, ab, tx, ct, sh, hw, tn, ot, dm, mf, dv, kf, fx, dq, bt, id, cc, nm, ox, px, rx, an, ui, sy] (2771)

15 9 or 10 or 11 or 12 or 13 or 14 (2177971)

16 1 or 2 or 3 (3414673)

17 5 or 6 or 7 (4626336)

18 15 and 16 and 17 (124162)

19 India.mp. [mp=ti, ab, tx, ct, sh, hw, tn, ot, dm, mf, dv, kf, fx, dq, bt, id, cc, nm, ox, px, rx, an, ui, sy] (754148)

20 18 and 19 (6323)

21 limit 20 to yr="2000 - 2021" (6105)

22 disability*.mp. [mp=ti, ab, tx, ct, sh, hw, tn, ot, dm, mf, dv, kf, fx, dq, bt, id, cc, nm, ox, px, rx, an, ui, sy] (987310)

23 disabled*.mp. [mp=ti, ab, tx, ct, sh, hw, tn, ot, dm, mf, dv, kf, fx, dq, bt, id, cc, nm, ox, px, rx, an, ui, sy] (223975)

24 persons with disability*.mp. [mp=ti, ab, tx, ct, sh, hw, tn, ot, dm, mf, dv, kf, fx, dq, bt, id, cc, nm, ox, px, rx, an, ui, sy] (1974)

25 22 and 23 and 24 (943)

26 Frail*.mp. [mp=ti, ab, tx, ct, sh, hw, tn, ot, dm, mf, dv, kf, fx, dq, bt, id, cc, nm, ox, px, rx, an, ui, sy] (156973)

27 Elderly*.mp. [mp=ti, ab, tx, ct, sh, hw, tn, ot, dm, mf, dv, kf, fx, dq, bt, id, cc, nm, ox, px, rx, an, ui, sy] (1433442)

28 26 and 27 (82093)

29 poor*.mp. [mp=ti, ab, tx, ct, sh, hw, tn, ot, dm, mf, dv, kf, fx, dq, bt, id, cc, nm, ox, px, rx, an, ui, sy] (4575717)

30 25 or 28 or 29 (4634572)

31 21 and 30 (3840)

***************************
