# Supplementary material for: Digital health interventions for improving access to primary care in India: A scoping review
Source: PLOS Glob Public Health. 2024 May 14;4(5):e0002645. doi: 10.1371/journal.pgph.0002645 (PMC11093327; doi:10.1371/journal.pgph.0002645)
Supplement: Attachment — Submitted filename: Response to reviewer comments.docx [file pgph.0002645.s004.docx]

**Response to reviewers’ comments:**

| Reviewer’s comments | Author’s response | Pages |
| --- | --- | --- |
| 1. The references seem to be mixed up and need correction. | We have made the necessary corrections after proof reading. | 28-33 (Entire reference Section) |
| 2. The use of the term 'assistive technology' for digital interventions in PHC is not common- Would you like to rephrase it to what is understood better in global health eg- 'digital health interventions' for access to PHC in vulnerable populations or something like that? | Thank you. We have revised the use of the term and have used digital health interventions as advised. | Entire Manuscript. |
| 3. Suggest to add / elaborate on what is unique about this scoping review, and why is it needed. | Thank you. We have included this section separately. Below the Introduction section,. | Page 7 |
| 4. Kindly consider a deeper analysis and critical appraisal of the 7 case studies | We completed a deeper analysis using MMAT tool and added that as a supplementary table previously. We have re-included it in the manuscript itself. | Page 23 |
| 5. NFHS 5 data is available now. Please include them in the introduction. | Thank you. We have included NFHS 5 data and corrected our reference/citation as well. | 4 & 28 |
| 6. Methods  It is good that the protocol of this coping review was registered with the Open Science Framework (OSF). Kindly provide the registration number.  “The search was first run on the 29th of November 2022 and was an updated search was run six months later on 30 April 2023”. It is actually a gap of 5 months! | The OSF protocol registration details can be accessed here - <https://osf.io/63pjw/>  It took sometime for us to start the screening process and hence a re-run of the search was completed. As recommended by Cochrane handbook – we will update the review in two years time. | Page 3. |
| 7. Utilization of Rayyan software for screening is mentioned only in the abstract. Please describe the details in the main article under methods section. | We have now added details about the screening conducted through Rayyan software | Page – 9 |
| 8. Was calibration exercise done at the step of selecting studies? If yes, how? and what did you find? Was inclusion criteria changed after the exercise? | We have described this aspect with further details. There was 80% between the independent screeners. We did not change the inclusion criteria. | Page - 10 |
| 9. A large number of studies were excluded. What are the reasons for exclusion? Please give details. | Yes we excluded a large number of studies because those studies did not fit the eligibility criteria for inclusion in this review. We have provided further details as a table below – if required we will include it in the manuscript. | No Changes made. |
| 10. As per the definition, assistive technologies include any device, product or service. But no studies about services are included in the review? | Thank you for the comment. We used this definition for our scoping review to begin with the search but we could not find any study with a pure service-related assistive technology. | No Changes made. |
| 11. Results 7 studies don’t represent all the regions of India? Provide the list of 12 Indian states in ENTraview - Shruti Tele otology program. | Thank you for the comment. We identified only seven studies related to our review objective from India. These the 7 studies include Uttar Pradesh, Bihar, Jharkhand and Karnataka, with some under-representation from north-east India. In the article related to Shruthi tele-otology program included for our scoping review*,* the authors do not specify which are the 12 states they included for their study of these 15 states where the Shruthi Program was active. | Page - 21 |
| 12. Conclusion Please avoid writing only recommendations under conclusion. Rather it should answer the research question “Can assistive technologies/ Digital health interventions for the vulnerable population improve access to primary care in India?” | Thank you for the kind input– we have modified the conclusion. | Page - 26 |

| **Reason for exclusion** | ***n (N = 3282)*** |
| --- | --- |
| Studies not relevant to the topic of scoping review or did not include all three key terms: ‘*Access to primary care’; ‘Vulnerable population’; and ‘Assistive Technology’* | 1367 |
| Conference abstracts | 290 |
| Review/systematic review/meta-analysis | 494 |
| Study designs not relevant to the topic | 339 |
| Prevalence studies | 187 |
| Surveys | 150 |
| Randomized controlled trials | 137 |
| Studies outside Indian context | 133 |
| Lab/animal studies | 124 |
| Conference presentations | 61 |
